# Supplementary material for: Chromothripsis during telomere crisis is independent of NHEJ, and consistent with a replicative origin
Source: Genome Res. 2019 May;29(5):737–49. doi: 10.1101/gr.240705.118 (PMC6499312; doi:10.1101/gr.240705.118)
Supplement: Supplemental Material [file supp_gr.240705.118_Supplemental_file_1.zip › contigs/annotated_contigs/DB89/contig.2.DB89_length_623_mean_cov_8.42696629213.docx]

**DB89_length_623_mean_cov_8.42696629213**

```
ATATTAAAATCATAGCCCAACATTTTTCAGGCAGTACCTCTGTATTCCCTTGAACTTAATATGCGTCTTGTCCAAACCTGGGTATTTAT
 >chr10:83930147-83930428 + E=2e-157 p=4e-02
TTTCTCCATGAAAATCCATATGGTTCACCATTCAACACCTGATTCTGAGTCTCAGCTTTTTCATTTATTAGTTGTTTGACCTTGGGCGA

AGTCTCACTAAAGCTTAATTTTCTCATTTTTAAAATGGGGAATAATTGTGAGAATTGAATGAGATGATGTTTATAAAACTTGTGGTATA

TATATATATATAT|A|CTCTGTATACTAGGAAAGGACATTAGTGGAAAACTCTAAGAAGGTTCCTCAAAAAATTAAAAATAGAATCACC
              >chrX:8962292-8962632 + E=7e-181
ATTTAATCCAGTAATGCCTCTTCTGGGTATATATTCAAAAAAACTAAAATCAGTATTCCCAAGAGAATCTTCACTGCCATGTTTATTGA

AGCATTATTATTCATAATCACTAACATATGGAAATAAATGAAATGTCCACAAATGAATAAAGAATACACACACACACACACACACACAA

ATATTATTTAACCATTAAAAATAAAATCCTGCCATTTGTAATAATGTAGATAATCCTGAAAAAAAATTATGTTAAGTAAGACAAATAA|

AAC
```
